# DeBakey Type II Acute Aortic Dissection Distant From the Proximal Edge of a Stent Graft: A Case Report Nine Years After Thoracic Endovascular Aortic Repair

**DOI:** 10.7759/cureus.50039

**Published:** 2023-12-06

**Authors:** Hideki Sasaki, Yoshiaki Sone, Shinji Kamiya, Yukihide Numata, Syunta Hayakawa

**Affiliations:** 1 Cardiovascular Surgery, Nagoya City University East Medical Center, Nagoya, JPN

**Keywords:** loss of consciousness, emergent operation, intimal tear, thoracic aneurysm, thoracic endovascular aortic repair, debakey type ii acute aortic dissection

## Abstract

An 84-year-old male with a medical history notable for prior thoracic endovascular aortic repair for thoracic aneurysm nine years ago presented to the emergency department with a chief complaint of transient loss of consciousness. A brain computed tomography showed no remarkable findings. A subsequent computed tomography scan for comprehensive evaluation revealed DeBakey type II acute aortic dissection as evidenced by contrast-enhanced imaging. An intimal tear was found on the ascending aorta distant from the proximal edge of a stent graft. Due to the urgency of the situation, the patient underwent emergent ascending aortic replacement. Following the successful intervention, the patient was transferred to a specialized rehabilitation facility with the goal of facilitating further improvement in their condition.

## Introduction

Type A acute aortic dissection (AAAD) is a life-threatening disease for which prompt diagnosis and treatment are necessary. An intimal tear can occur at any part of the aorta, from the ascending aorta to the descending aorta. In clinical settings, the ascending aorta is often where the intimal tear is found in AAAD. In an aging society, patients presented to the surgeon tend to have more comorbidities and complex past medical histories. Meanwhile, thoracic endovascular aortic repair (TEVAR) has gained popularity due to the pursuit of minimally invasive treatment. While it is beneficial to patients in terms of minimal invasiveness, leading to early recovery in the short term, complications can be more complex in the long term. Physicians have to pay attention to issues such as retrograde type A aortic dissection (RTAD) associated with TEVAR, as well as unexpected situations [1,2]. Herein, we present a case of DeBakey type II acute aortic dissection in which an intimal tear was found at a site distant from the proximal edge of a stent graft.

## Case presentation

**Figure 1:**
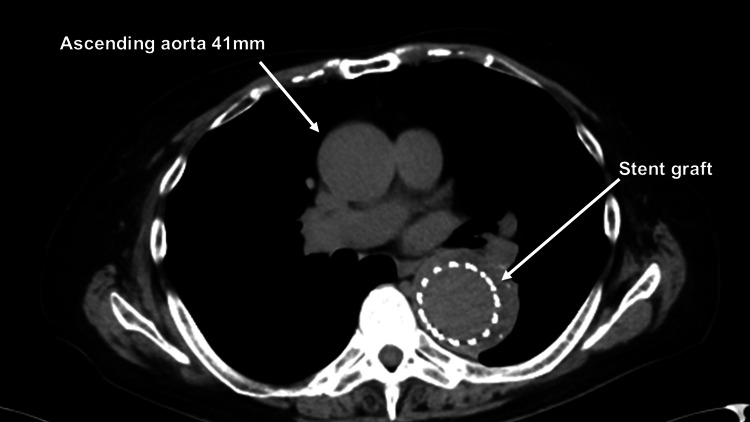
Computed tomography nine months earlier A computed tomography scan nine months earlier revealed that the diameter of the ascending aorta is 41 mm.

An 84-year-old male presented to the emergency department with a transient loss of consciousness. The patient underwent TEVAR (COOK Zenith TX2 42 mm) for a descending thoracic aneurysm nine years ago and has been regularly followed in the outpatient clinic. At the last visit nine months ago, the diameter of the descending thoracic aneurysm showed no change, and there was no migration of the stent graft. The diameter of the ascending aorta was 41 mm (Figure 1).

**Figure 2:**
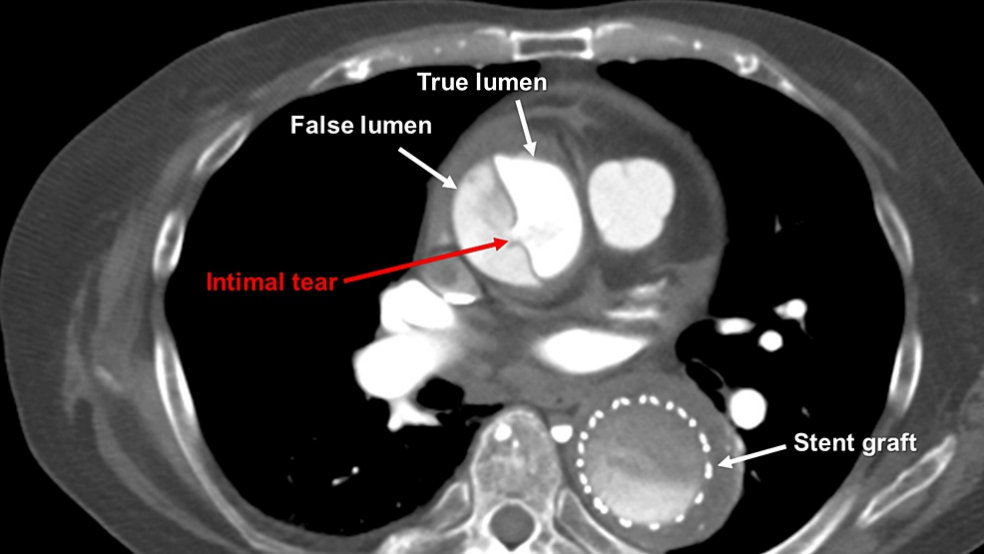
Preoperative computed tomography A preoperative computed tomography scan revealed an intimal tear at the ascending aorta.

In the emergency department, the patient was alert with no apparent paralysis. Head computed tomography revealed no remarkable findings. Emergent contrast-enhanced computed tomography revealed AAAD, with the extent of dissection ranging from the sinotubular junction to the proximal aortic arch. An intimal tear was identified at the ascending aorta (Figure 2), leading to the diagnosis of DeBakey type II acute aortic dissection.

**Figure 3:**
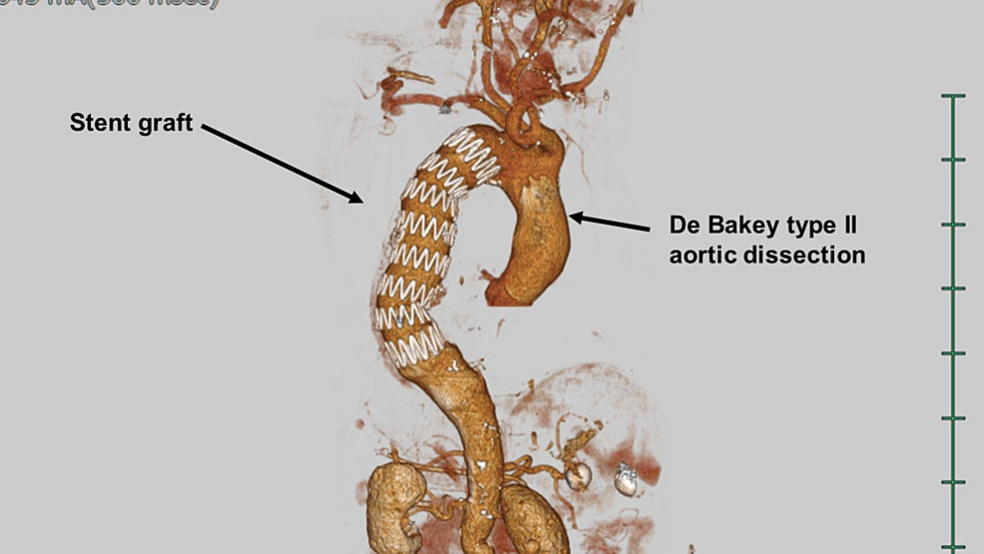
Preoperative computed tomography image A preoperative computed tomography scan showed no aortic dissection at the aortic arch.

**Figure 4:**
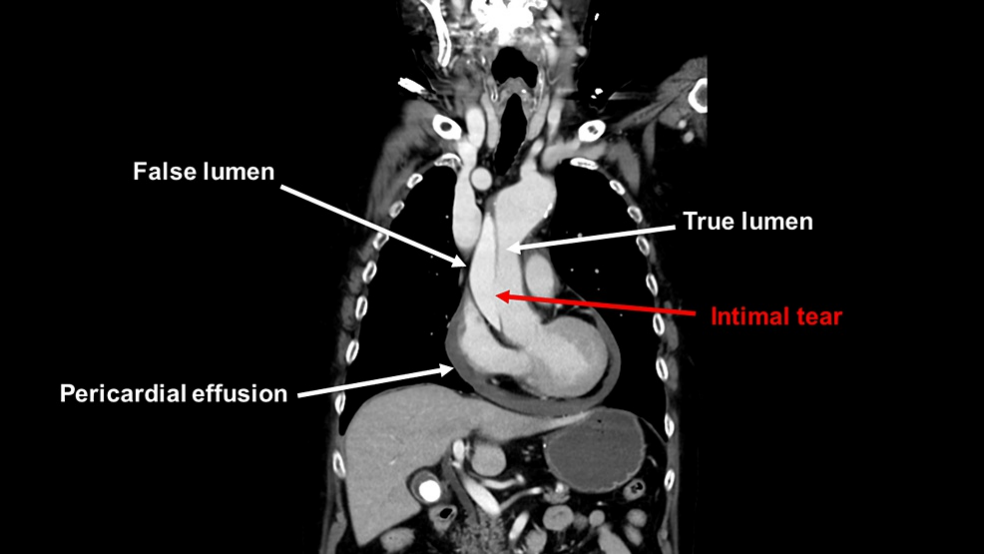
Preoperative computed tomography image A preoperative computed tomography scan showed an intimal tear located at ascending aorta and pericardial effusion.

No intimal tear was found at the proximal edge of the stent graft (Figures 3, 4).

After a thorough discussion between cardiovascular surgeons and radiologists, we decided to perform emergent surgery. Under general anesthesia, the patient was positioned in the supine position. A median sternotomy was performed, and cardiopulmonary bypass was established with left femoral artery perfusion and vena cava drainage. The patient was cooled down to a rectal temperature of 25 °C. During circulatory arrest, the ascending aorta was opened, revealing a large intimal tear above the sinotubular junction. Although the dissection extended to the proximal arch, no other tears were found around the proximal edge of the stent graft.

Retrograde cerebral perfusion and retrograde cardioplegia were initiated. The decision was made to perform ascending aortic graft replacement. A branched graft (Triplex 26 mm, Terumo Corp, Tokyo, Japan) was selected and anastomosed to the proximal arch, restoring systemic perfusion. After the distal anastomosis, the graft was connected to the proximal ascending aorta. Upon completion of all anastomoses, the cross-clamp was released. The heart resumed spontaneous sinus rhythm, and the patient was successfully weaned from cardiopulmonary bypass without difficulty.

**Figure 5:**
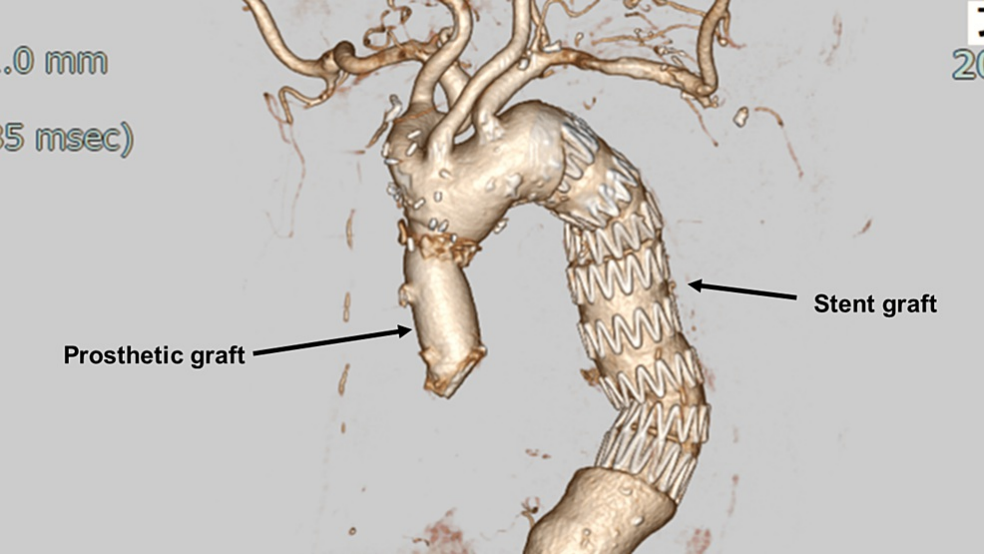
Postoperative computed tomography image A postoperative computed tomography scan revealed no dissection at the aortic arch.

The patient was transferred to the intensive care unit in stable condition and extubated on postoperative day 1. Subsequently, the patient was transferred to the ward in stable condition. Contrast-enhanced computed tomography revealed no residual dissection (Figure 5). The patient was then transferred to a rehabilitation facility for further recovery.

## Discussion

The strategy for thoracic aortic diseases, including aortic dissection and degenerative aneurysm has evolved since the emergence of TEVAR [3]. TEVAR has been widely accepted as a less invasive surgical option, particularly beneficial for elderly patients who often present with comorbidities. However, physicians overseeing patients who have undergone TEVAR must vigilantly monitor the occurrence of adverse events associated with TEVAR, both in the short and long term. RTAD and endoleak are notable among these events [4,5]. Although AAAD occurred in the current case, it deviated from our initial predictions before contrast-enhanced computed tomography revealed the condition. We have identified four key points for discussion in this case.

The first point is the observation that an intimal tear can occur at any site in the aorta, regardless of TEVAR. Age is a crucial factor contributing to various diseases. In this case, the patient had undergone TEVAR for a descending thoracic aneurysm, prompting us to maintain regular surveillance for the stent graft's position and the aortic diameter. This monitoring aims to detect potential dilatation of the descending thoracic aneurysm due to endoleak. Simultaneously, we have been monitoring the diameter of the ascending aorta and aortic arch. While our case did not experience a rapid expansion of the aortic diameter, AAAD occurred nine years after TEVAR. This case underscores the difficulty in predicting the onset of AAAD, even with regular physician monitoring of aortic diameter.

The second point concerns the unexpected location of the intimal tear in this patient. The entry point was situated in the ascending aorta, and the extent of dissection was limited to the ascending aorta, classifying it as DeBakey type II aortic dissection. The aortic arch was not involved, and a stent-graft-induced new entry tear (SINE) was not identified. When informed about this case by the emergency department physician before the computed tomography scan, our initial suspicion was RTAD. However, this was not the case for our patient. In clinical settings, past medical history can significantly influence the diagnostic process. Typically, when cardiovascular surgeons are consulted regarding such a patient, they might lean towards considering RTAD as a possibility, and such an inference may seem reasonable. However, this case emphasizes the importance of excluding bias and conducting an accurate diagnosis based on physical examinations and imaging studies. The degenerative changes in the aorta, which often accompany aging, may have contributed to the development of AAAD in this particular case.

The third point relates to the size of the aorta. A diameter of 41 mm is relatively large, attracting a physician's attention. However, it does not meet the criteria for surgical intervention. Regular surveillance, once per year, is deemed reasonable as long as the diameter remains stable over the years.

The fourth point concerns the choice of the procedure. Although arch vessels were not involved in the current case, an alternative option is ascending aorta and aortic arch replacement with the reconstruction of arch vessels. If the patient were younger, considering the potential for future dilatation of the aortic arch, arch replacement would be a reasonable option. The choice of procedure should be made on a case-by-case basis, taking into account the patient's age, comorbidities, and malperfusion associated with AAAD.

## Conclusions

In this study, DeBakey type II acute aortic dissection manifested nine years following the performance of TEVAR for a descending thoracic aneurysm. Notably, no discernible intimal tear was identified around the proximal edge of the stent graft. This case emphasizes the critical importance of accurate diagnosis grounded in thorough physical examinations and imaging studies while actively mitigating biases in clinical assessments.
